# Retraction: Biomarkers for Presymptomatic Doxorubicin-Induced Cardiotoxicity in Breast Cancer Patients

**DOI:** 10.1371/journal.pone.0219887

**Published:** 2019-07-11

**Authors:** The PLOS ONE Editors

Following publication of this [1] article, the corresponding author notified the journal at the request of the Central Arkansas Veterans Health Services (CAVHS) Associate Chief of Staff for Research and Development that part of the human subjects research described in the article did not receive approval from the CAVHS Institutional Review Board.

As stated in the article, the authors received approval from the University of Arkansas for Medical Sciences (UAMS) Institutional Review Board (UAMS IRB 2010 approved protocol #130212) where patients were recruited, consented, and participated. However, analysis of the specimens occurred, in part, at CAVHS. The specimens were not de-identified, and thus required approval from the CAVHS IRB prior to initiating this work. The authors explained that they were unaware of the requirements at the time. The CAVHS Chief of Staff and IRB agreed that the investigators had received inadequate education about the ethics approval requirements, and both the study and the program were determined to be non-compliant.

Owing to lack of required IRB oversight for the research reported in this article, the *PLOS ONE* Editors retract this article.

All authors did not agree with retraction.
